# The Spiritual Well-Being Scale in the Polish Catholic Setting: Construct, Structure, and Relationships with Psychological Well-Being

**DOI:** 10.1007/s10943-024-02138-w

**Published:** 2024-10-06

**Authors:** Beata Zarzycka, Kamil Tomaka, Michał Grupa, Raymond F. Paloutzian, Rodger K. Bufford

**Affiliations:** 1 Institute of Psychology, The John Paul II Catholic University of Lublin, Aleje Raclawickie 14, 20-950 Lublin, Poland; 2 Department of Psychology, Westmont College, Santa Barbara, USA; 3 Graduate School of Clinical Psychology, George Fox University, Newberg, USA

**Keywords:** Spirituality, Religion, Spiritual well-being scale, SWBS, Psychometrics

## Abstract

The population of Poland could be of particular interest to researchers studying religion and spirituality. Catholicism has played a significant role in shaping the country’s culture, history, and tradition and is considered a national religion. More recently, however, the processes of moving away from regular participation in religious services and loosening ties with the institution of the Catholic Church are marked (Polish Public Opinion Research Center, CBOS). The Polish Catholic setting may modify the associations between psychological variables that have been studied in their relationships with spirituality, which have been investigated in religious contexts differing from a Polish Catholic one. Given this context, we examined the internal structure of spiritual well-being in the sample of present-day Polish Catholics (Study 1) and how spiritual well-being is related to mental health (Study 2). The internal structure of the Spiritual Well-Being Scale was replicated in the Polish sample, but only positively worded items loaded on the Religious and Existential Well-Being subscales. Spiritual well-being was positively related to positive mood, satisfaction with life, and psychological well-being; it was negatively associated with negative mood, depression, and anxiety, thus remaining significantly related to psychological well-being among Polish Catholics.

**Supplementary Information:**

The online version contains supplementary material available at 10.1007/s10943-024-02138-w.

## Introduction

Spiritual well-being has been identified as an essential aspect of subjective psychological well-being (Ellison, 1983). The construct of spiritual well-being (SWB) includes a religious and an existential component. The former refers to a sense of well-being in a relationship with God. The latter relates to the perception of purpose and satisfaction with life. Both components, or dimensions, contribute to people’s overall psychological well-being, and are often more helpful than objective, economically oriented indicators (Ellison, 1983; Moberg, 1979; Paloutzian & Ellison, 1982). Developments in the fields of religion, psychology, and spirituality have demonstrated that these two dimensions have functional relationships with physical and mental health. Paloutzian and Ellison (1982) developed the Spiritual Well-Being Scale (SWBS), which measures SWB and is comprised of the religious well-being (RWBS) and existential well-being (EWBS) subscales.

Initially, most of the studies conducted with the SWBS included evangelical and Protestant populations (Scott et al., 1998). But in recent years, the research has expanded to include other religious groups, such as Muslims (Musa, 2016; Musa & Pevalin, 2014), diverse cultural contexts (Bufford & Paloutzian, 2023/4), various ethnic groups (Miller et al., 1998), and secularized societies (Malinakova et al., 2017; Tavel et al., 2021). These studies have provided essential data on the construct of SWB and the instrument developed to measure it. Importantly, the structure of the SWBS may differ depending on cultural and denominational backgrounds because religion and spirituality are variables conditioned by both cultural and denominational traditions (Miller et al., 1998; Musa, 2016; see Bufford & Paloutzian, 2023/4, and Paloutzian et al., 2021, for comprehensive presentations of SWBS research in different countries and languages). Therefore, further progress on SWBS research in Poland necessitated that we explore its statistical and psychological properties while considering different cultural and religious backgrounds.

Poland is an area that could be of particular interest to researchers studying religion and spirituality. Catholicism has significantly shaped the country’s culture, history, and traditions and is considered a national religion (Zarzycka et al., 2023). Most Polish people declare that they belong to a Christian denomination, especially Catholicism. Self-declaration rates of faith have remained consistently high for several decades. At the same time, however, the way Polish people believe is changing. Above all, the processes of moving away from regular participation in religious services and loosening ties with the institution of the Catholic Church are clearly marked (Polish Public Opinion Research Centre, CBOS, 2022). Because one’s setting and context may modify the associations between spirituality and other psychological variables, the present study’s first aim was to analyze the internal structure of the SWBS in a present-day Polish Catholic sample. The second aim was to examine how SWBS scores relate to mental health. Specifically, we investigated positive and negative mood, satisfaction with life, anxiety, depression, and psychological well-being as indicators of mental health.

Our study contributes to the existing literature in several ways. First, given the results of previous studies, it provides knowledge about a construct that is validated and important in spirituality research, although not previously analyzed in a culturally Catholic religious context. Second, this research provides knowledge about the differential relevance of the SWBS to other widely used measures of psychological well-being and quality of life.

## Spiritual Well-Being

According to Campbell (1981), psychological well-being is associated with the satisfaction of three basic needs: the need to have purpose, the need to belong and experience intimacy, and the need to feel satisfied with oneself. Ellison (1983) identified the need for transcendence as the fourth factor contributing to well-being. This refers to “the sense of well-being that we experience when we find purposes to commit ourselves to which involve ultimate meaning for life” (Ellison, 1983, p. 330). Since this feeling relates to the non-physical dimension of consciousness and experience, it has been called spiritual.

The definition of spiritual well-being (SWB) includes a religious and socio-psychological component (Ellison, 1983). It draws on Moberg’s (1979) distinction between a vertical and horizontal dimension of well-being. The former refers to a sense of well-being in a relationship with God. The latter has no religious references but instead evokes existential references that relate to the perception of purpose and satisfaction with life. Both dimensions involve transcendence or leaning out and beyond and reflect how people describe what SWB means to them (Paloutzian & Ellison, 1982). SWB is not synonymous with spirituality but is an outcome variable, a construct that accompanies spirituality. Nor can high SWB be identified with spiritual maturity, although we would expect a spiritually mature individual to have a positive sense of SWB (Ellison, 1983). Finally, SWB is not synonymous with spiritual health, but is instead a condition that underlies spiritual health and quality of life (Bufford et al., 1991; Ellison, 1983). SWBS scores should be considered a continuous variable, not a dichotomous one. It is not a question of whether we have it or not, but of degree, that is, of how much we may increase, decrease, or experience our SWB (Ellison, 1983).

## Spiritual Well-Being Scale

Paloutzian and Ellison (1982) developed the 20-item Spiritual Well-Being Scale (SWBS) to measure one’s perception of SWB, including the religious and existential dimensions. The Religious Well-Being subscale (RWBS) contains ten items that reflect well-being in traditionally religious language, focusing on one’s relationship with God. The Existential Well-Being subscale (EWBS) includes ten items that reflect well-being in a religious-existential language, measuring life purpose and satisfaction (Paloutzian & Ellison, 1982). The RWBS and EWBS scores are summed to yield the total SWBS score, which equals the sum of the responses to all twenty items. The content of the items does not address specific theological issues or a-priori standards of well-being, which may vary depending on the religious belief system or denomination (Paloutzian & Ellison, 1982). Each item is answered on a 6-point Likert scale ranging from 1 (*strongly disagree*) to 6 (*strongly agree*); high scores indicate more well-being. About half of the items indicate the absence of well-being and are reverse-scored. The SWBS has demonstrated good internal consistency and construct validity, although some differences are noted across languages and cultural settings (Bufford et al., 1991; Bufford & Paloutzian, 2023/4; Paloutzian et al., 2021).

There are certain exceptions to using all SWBS items to get the most precise measurement of SWB and its components for a given sample or population. As will be seen in research presented below, in some instances due to language, cultural, or other differences, not all twenty items function to make for the best measure. In these cases, calculating and comparing mean item scores better facilitates comparisons across samples. Even so, the general trends across such datasets can be compared with the analogous trends in other research that has used all twenty items. These variations will become clearer as they are illustrated below with both a 20-item and an 11-item version of an SWBS in Polish.

Although the SWBS “has become a standard bearer in the religious and spiritual well-being literature …” (Hill, 2013, p. 59), the instrument is not without psychometric problems. Some studies have shown that SWBS items are not consistently organized into the assumed two factors (Miller et al., 1998; Musa, 2016; Musa & Pevalin, 2014; Scott et al., 1998). For example, Scott et al. (1998) found a three-factor structure in a clinical sample of psychiatric inpatients. The factors were labeled Affiliation, Alienation, and Dissatisfaction with Life. Miller et al. (1998) found a three-factor structure (Religious Well-Being, Life Satisfaction/Purpose, Future) for Caucasian participants and a five-factor structure (Connection with God; Satisfaction with God and Day-to-Day Living; Future /Life Contentment; Personalized Relationship with God; Meaningfulness) for African Americans. The Arabic version of the SWBS for the Jordanian Arab Christians (Musa & Pevalin, 2014) and the Malaysian Muslims (Musa, 2016) also proved to have a three-factor structure (Positive Existential Well-Being, Affiliation, and Alienation).

Since the construct validity of the SWBS, as established by factor analysis, is still far from conclusive, a number of recommendations were made for further research (Genia, 2001; Miller et al., 1998). First, it is important to understand that, technically speaking, factors are not properties of scales; they are properties of datasets. The need for further studies using exploratory and confirmatory factor techniques was suggested to identify components of the SWBS (Ledbetter et al., 1991). Second, the need to assess the utility of the scale for Catholics, Jews, and believers outside the Judeo-Christian tradition was pointed out (Ellison, 1983). Finally, the need to recruit more heterogeneous samples was suggested, as the tool is highly negatively skewed in the religious population (Bufford et al., 1991; Ledbetter et al., 1991).

There is little research in non-Western samples, especially involving Christian denominations other than evangelical Protestants. In Europe, relatively recent studies were conducted in the Czech Republic with a representative sample of adults (Tavel et al., 2021) and adolescents (Malinakova et al., 2017). A two-factor solution was confirmed for the SWBS in the adult sample, with factors corresponding to the Religious and the Existential subscales when negatively worded items were excluded from the scale. Problems with negative items also manifested in that the 20-item SWBS showed low internal consistency, whereas after excluding negatively worded items, it showed good values (Tavel et al., 2021). Psychometric evaluation of a shortened version of the SWBS in a sample of Czech adolescents led to similar conclusions. It confirmed the two-factor structure after removing the negatively worded items (Malinakova et al., 2017). The Czech Republic has the highest percentage of religiously unaffiliated people in the world (Tavel et al., 2021), which may help explain the different structure of its SWBS datasets compared to those of other cultural contexts. Nevertheless, the results observed so far indicate the relevance of considering the cultural context for understanding SWBS items (Miller et al., 1998). Bufford and Paloutzian (2023/4) proposed that in some language and cultural settings, reversely worded items are not responded to consistently and can be described as a method factor.

Poland is an area of particular interest in religiousness and spirituality research because it is a country with consistently high rates of religious faith. The percentage of people identifying as believers has consistently exceeded 90% in Poland since the late 1990s. However, when the frequency of attendance at religious services is analyzed, a slow decline in the number of those attending weekly and a slow increase in the percentage of those attending irregularly or not at all (Polish Public Opinion Research Center, CBOS, 2020) is visible. These data show that Polish citizens, like other members of Western societies, are becoming more culturally Catholic, more loosely tied to religious institutions, and more individualized in expressing their religiousness. Such a religious setting may provide a good background for research on SWB. The present study aimed to analyze the internal structure of the Polish version of the SWBS and evaluate its relationship with psychological well-being.

When translating the SWBS from English to Polish, we followed the guidelines by Koenig and Al Zaben (2021). Before we started the translation work, we obtained the approval of one of the scale developers (RFP), who was invited to participate in the translation process. Three translators independently translated the items into Polish. The translators were native speakers of Polish, born and raised in Polish culture, and currently residing in Poland. They were also fluent in English and had a background in psychology. A translation panel consisting of a principal investigator (PI) and three translators prepared an accepted Polish version of the SWBS. This was back-translated by three bilingual Polish graduate students with a background in linguistics and training in psychological assessment. After the back-translation, the translators met with the PI to reach a consensus on the back-translation. The back-translation was reviewed by the scale developer (RFP). Identified discrepancies were discussed with the PI and resolved by correcting the translated version. The scale developer approved the back-translated version. To ensure that the translated scale was comprehensible to the target population, several adults, male and female, native speakers of Polish, were interviewed. Any minor language errors were corrected in the final version.

### Study 1. The Internal Structure of the SWBS

Study 1 aimed to examine the internal structure and psychometric properties of the Polish SWBS. To examine its dimensionality, we followed Koenig and Al Zaben (2021) criteria for psychometric validation and translation of religious and spiritual measures. We also established sociodemographic differences in SWBS subscale scores.

#### Method

##### Participants

Two samples of adult Poles allowed us to collect data for exploratory (EFA, Sample 1) and confirmatory (CFA, Sample 2) factor analysis.

To determine the sample size for the EFA, we followed the recommendations by Kyriazos (2018). The required minimum number of participants should be between 100 and 300, and the ratio of participants to items not less than 5:1. Thus, sample 1 included 318 adults (51.9% women) aged 18 to 60 years (*M* = 39.66, *SD* = 12.44). Most (*n* = 267, 84.0%) declared themselves Catholics. The others declared affiliation with other Christian denominations (e.g., Greek Catholicism, *n* = 3, 0.9%; Protestants, *n* = 2, 0.6%; Orthodoxy, *n* = 1, 0.3%), and others (*n* = 5, 1.5%). The remaining 12.6% (*n* = 40) of participants reported no religious affiliation. The majority (*n* = 216, 58.2%) of the respondents said they were somewhere between high and low in religiousness. Specifically, a small number said they were definitely religious (*n* = 12, 3.8%), some moderately religious (*n* = 111, 34.9%), and some religious (*n* = 62, 19.5%). Some reported that they were non-religious (*n* = 33, 10.4%), some moderately non-religious (*n* = 57, 17.9%), and some definitely non-religious (*n* = 43, 13.5%). Most participants (*n* = 235, 73.9%) reported living in urban areas and completed at least secondary education (*n* = 284, 89.3%).

The recommended sample size for CFA is 5–20 individuals for each parameter estimate (Schumacker & Lomax, 1996). There were 63 parameters to be estimated, and thus, we collected data from 303 adults (54.1% women) aged between 18 and 60 years (*M* = 39.17, *SD* = 12.40). Most of them (*n* = 229, 75.3%) declared themselves to be Catholics. The remaining respondents represented other Christian denominations (*n* = 14, 4.6%; e.g., Greek Catholicism *n* = 6, 2.0%); The final 17.5% (*n* = 54) of participants reported no religious affiliation. In order of religiousness, the respondents described themselves as definitely religious (*n* = 17, 5.6%), moderately religious (*n* = 86, 28.4%), religious (*n* = 70, 23.1%). Some reported that they were non-religious (*n* = 27, 8.9%), some moderately non-religious (*n* = 58, 19.1%), and some definitely non-religious (*n* = 45, 14.9%). Most of the respondents (*n* = 224, 73.6%) reported living in urban areas and having completed at least secondary education (*n* = 272, 89.5%). The more detailed characteristics of respondents of Samples 1 and 2 are presented in Table S1 (see Supplementary Material).

##### Procedure

We used non-probabilistic convenience sampling to select the participants. All participants were users of the Ariadna online research panel. We aimed to have a religiously heterogeneous sample. Thus, we would avoid the ceiling effect (negative skewness) that appeared in SWBS studies in samples of very religious people (Bufford et al., 1991; Genia, 2001; Ledbetter et al., 1991; Scott et al., 1998). The data were collected through an online survey. Anonymity was ensured, and informed consent was obtained from all participants. The procedure was approved by the Research Ethics Committee at the Institute of Psychology at the first author’s university.

##### Data Analysis

Prior to conducting the analysis, we explored the database to identify atypical cases or missing values based on Mahalanobis distance. There were no atypical and missing values in the data set. To explore the internal structure of the Polish SWBS we performed a PCA, followed by varimax rotation in Sample 1 (*n* = 318) (e.g., Ellison, 1983; Miller et al., 1998). Because some authors (e.g., Ledbetter et al., 1991) argued that it is more appropriate to use oblique rotation (because there is some correlation between the RWB and EWB subscales), we also checked the structure of the Polish SWBS using PCA with oblimin rotation (Malinakova et al., 2017). We established a factor loading > 0.40, an alternative factor loading < 0.30, and a difference of at least 0.20 between the primary and alternative factor loadings as criteria for factors’ item retention, thus omitting those items that did not meet these criteria (Howard, 2016). To confirm the internal structure of the PSWBS, we conducted CFA using AMOS (Arbuckle, 2019). We performed CFA on Sample 2 (*n* = 303) using the maximum likelihood estimation method. To evaluate the goodness of fit of the model, we used the following statistics: χ^2^, the Tucker-Lewis index (TLI), the comparative fit index (CFI), the normed fit index (NFI), the standardized root mean square residual (SRMR), and the root mean squared error of approximation (RMSEA) with the 90% confidence interval (CI). Then, we calculated descriptive statistics, skewness, and kurtosis. The normality of the data distribution was assessed using histograms and then tested using the Shapiro–Wilk test. Cronbach’s *α* was used to establish the internal consistency of the Polish SWBS.

##### Measures

Three translators translated the SWBS from English to Polish. It was then back-translated by three bilingual Polish graduate students with a linguistics background and psychological assessment training. A committee of two bilingual assistants with doctoral degrees in psychology made the final selection of items for the Polish SWBS. One of the co-authors of the SWBS [RFP] examined and approved the item wording.

###### Spiritual Well-Being

The initial Polish SWBS consisted of 20 items measuring participants’ perception of the spiritual quality of life (Paloutzian & Ellison, 1982). The response options ranged from 1 (*strongly disagree*) to 6 (*strongly agree*). The Polish SWBS has two subscales: Religious Well-Being (RWB, e.g., *I believe that God loves me and cares about me*) and Existential Well-Being (EWB, e.g., *I feel that life is a positive experience*). All RWBS items contain the Polish word for “God”. None of the EWBS items contain it. A combined Polish SWBS total score provides a general measure of SWB in Polish samples. Approximately half of the items are negatively worded and have reverse scoring to control for response-set bias, so that disagreement with these items reflects higher well-being. Thus, with these scores added to the responses to the positively worded items (for which higher agreement was given a higher score), the Polish SWBS total score is provided, which yields a score that can range from a low of 20 (reflecting low well-being) to a high of 120 (reflecting high well-being).

### Results

#### Exploratory Factor Analysis

In Sample 1, the mean scores for the Polish SWBS items ranged from 2.94 (Item 4) to 4.34 (Item 18), with a grand mean of 3.61 (*SD* = 0.47). Factor analysis met the standards of the KMO measure of sampling adequacy (Kaiser, 1970) and the Bartlett Test of Sphericity (KMO = 0.89; Bartlett’s χ^2^ (190) = 3630.87, *p* < 0.001), indicating that items were sufficiently intercorrelated, so factor analysis was appropriate. Based on Kaiser’s criterion (eigenvalue higher than 1), three factors needed to be distinguished, explaining 59.49% of the variance (Table 1). Factor 1 included all the items measuring RWB. However, although three of the four negatively worded items (1, 5, 9, and 13) received the highest factor loadings in F1, they also entered F3. The factor loadings for these items in F3 were ≥ 0.36 and differed by ≤ 0.21 from their loadings on F1. The items measuring EWB split and entered into two separate factors. All positively worded items (PWI) were included in Factor 2, and all negatively worded items (NWI) were included in Factor 3. Item 16, although it achieved the highest factor loading in F3, achieved a factor loading higher than 0.30 in F2 as well. In conclusion, the EFA with varimax rotation showed a three-factor structure for the Polish SWBS. However, the suggested solution cannot be considered satisfactory as five items were cross-loaded on two factors. All cross-loadings involved negatively worded items, four of which belonged to the RWBS; see Table 1.

**Table 1:** Exploratory Factor Analysis Showing 20 Items and Factor Loadings from the Pattern Matrix (Principal Component Analysis with Varimax Rotation) in Sample 1

Items		Factor loadings		
		**F1**	**F2**	**F3**
19	Moja relacja z Bogiem pomaga mi osiągnąć dobre samopoczucie [My relation with God contributes to my sense of well-being]	**0.89**	0.20	0.07
17	Czuję się bardzo spełniony, gdy jestem w bliskiej komunii z Bogiem [I feel most fulfilled when I’m in close communion with God]	**0.88**	0.20	0.06
15	Dzięki mojej relacji z Bogiem nie czuję się osamotniony [My relationship with God helps me not to feel lonely]	**0.87**	0.19	− 0.03
11	Wierzę, że Bóg interesuje się moimi problemami [I believe that God is concerned about my problems]	**0.87**	0.25	0.04
7	Mam osobistą, głęboką relację z Bogiem [I have a personally meaningful relationship with God]	**0.81**	0.27	0.09
3	Wierzę, że Bóg mnie kocha i troszczy się o mnie [I believe that God loves me and cares about me]	**0.71**	0.24	− 0.03
13*	Nie odczuwam osobistej satysfakcji z mojej relacji z Bogiem [I don’t have a personally satisfying relationship with God]	− **0.64**	0.05	**0.43**
9*	Bóg nie jest dla mnie znaczącym źródłem osobistej siły i wsparcia [I don’t get much personal strength and support from my God]	− **0.49**	0.23	**0.40**
5*	Uważam, że Bóg jest bezosobowy i nie interesuje się moimi codziennymi sprawami [I believe that God is impersonal and not interested in my daily situations]	− **0.49**	0.15	**0.46**
1*	Osobista modlitwa do Boga nie daje mi wiele satysfakcji [I don’t find much satisfaction in private prayer with God]	− **0.47**	0.14	**0.36**
8	Czuję się spełniony i zadowolony z życia [I feel very fulfilled and satisfied with life]	0.13	**0.83**	− 0.17
10	Odczuwam zadowolenie, gdy myślę o kierunku, w jakim podąża moje życie [I feel a sense of well-being about the direction my life is headed in]	0.17	**0.83**	− 0.09
14	Jestem spokojny o moją przyszłość [I feel good about my future]	0.17	**0.72**	− 0.14
20	Uważam, że moje życie naprawdę ma jakiś cel [I believe there is some real purpose for my life]	0.21	**0.68**	− 0.25
4	Życie jest dla mnie pozytywnym doświadczeniem [I feel that life is a positive experience]	0.11	**0.66**	− 0.25
12*	Niewiele w życiu mnie cieszy [I don’t enjoy much about life]	0.09	− 0.38	**0.69**
6*	Moja przyszłość sprawia wrażenie niepewnej [I feel unsettled about my future]	− 0.02	− 0.16	**0.66**
18*	Życie nie ma większego znaczenia [Life doesn’t have much meaning]	− 0.01	− 0.26	**0.64**
2*	Nie wiem kim jestem, skąd przybyłem, ani dokąd zmierzam [I don’t know who I am, where I came from, or where I’m going]	− 0.09	− 0.12	**0.58**
16*	Czuję, że życie jest pełne konfliktów i nieszczęść [I feel that life is full of conflict and unhappiness]	0.13	− **0.32**	**0.54**
	Eigenvalues	5.53	3.54	2.83
	% of variance	27.63	17.70	14.16
	Cumulative variance	27.63	45.33	59.49

Items for each factor are listed in descending order based on loadings. * The item is negatively formulated. The boldfaced text indicates items assigned to each factor. F1-Religious Well-Being, F2-Existential Well-Being (EWB +), F3-Existential Well-Being (EWB-).* N* = 318

Spiritual Well-Being Scale copyright 1982 C. W. Ellison & R. F. Paloutzian. Translation into Polish courtesy of Professor Beata Zarzycka. Copyright 2024 R. F. Paloutzian. For more information see https://www.westmont.edu/psychology/raymond-paloutzian-spiritual-wellbeing-scale

#### Confirmatory Factor Analysis

In Sample 2., the means of the Polish SWBS items ranged from 2.93 (Item 4) to 4.36 (Item 18), with a grand mean of 3.63 (*SD* = 0.79). We used CFA to test four models based on the results of previous studies (e.g., Ledbetter et al., 1991; Tavel et al., 2021). The first is a one-dimensional model. The high correlations between subscales suggest that the SWBS can be conceptualized as assessing a general single-factor construct that describes one dimension of well-being (Ledbetter et al., 1991). The second is a two-factor model (EWBS and RWBS) in which the ten RWBS items and ten EWBS items are directly affected by the RWBS and the EWBS latent constructs, respectively, in accordance with the theoretical background of the SWBS (Ellison, 1983). Some prior studies have not supported either the one-factor or 2-factor model (e.g., Bufford & Paloutzian, 2023/4; Ledbetter et al., 1991), suggesting that the organization of SWBS items can be more complex. Therefore, we tested a three-dimensional model (Polish RWB, EWB + , and EWB–), which we obtained in the EFA, and finally, a two-dimensional model (Polish RWB and EWB) containing only positively worded items (Tavel et al., 2021). Table 2 includes the fit indices of the models we tested; see Table 2.

**Table 2:** Summary of Confirmatory Factor Analysis on the Polish Spiritual Well-Being Scale in Sample 2 (n = 303)

	Model	χ^2^	*df*	TLI	CFI	NFI	SRMR	RMSEA [90% CI]
1	One-Dimensional Model	1401.83	170	0.618	0.658	0.630	0.153	0.155 [0.147, 0.162]
2	Two-Dimensional Model (EWB, RWB, all items included)	710.03	169	0.831	0.850	0.813	0.085	0.103 [0.095, 0.111]
3	Three-Dimensional Model (RWB, EWB + , EWB–)	608.25	167	0.861	0.877	0.840	0.082	0.093 [0.085, 0.101]
4	Two-dimensional (RWB + , EWB +)	125.14	43	**0.960**	**0.968**	**0.953**	**0.051**	**0.079** [**0.063**, **0.096**]

All chi-squared values were statistically significant at the *p* < .001 level; goodness-of-fit measures in bold indicate the satisfactory fit. *N* = 303

**Fig. 1:**
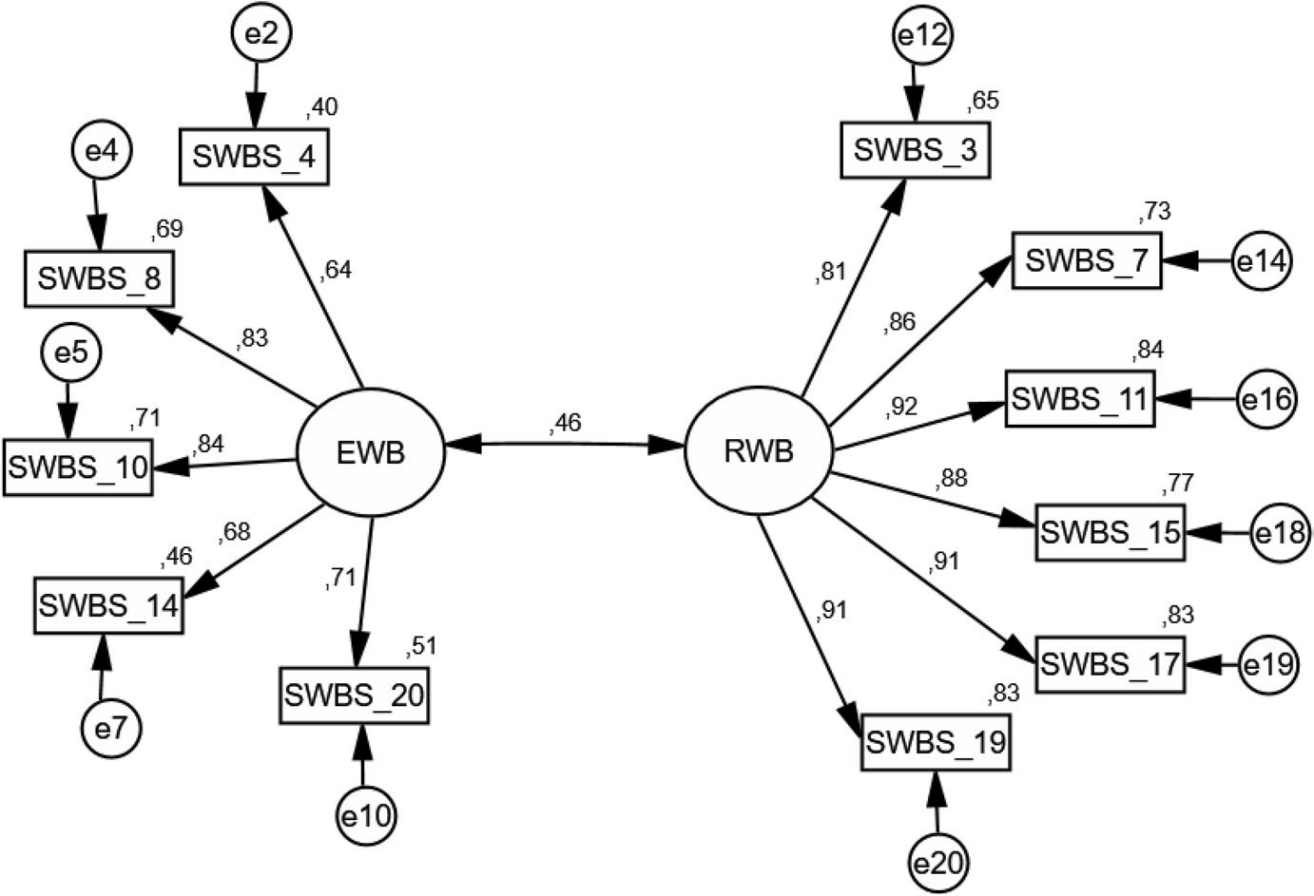
Confirmatory Factor Analysis of the Polish Spiritual Well-Being Scale (two-dimensional model, including only positive formulated items)

Model 4, two-dimensional, including only positively formulated items, obtained the best fit. The χ^2^ value was 125.14, which at 90% CI resulted in a significance level of *p* < 0.001; thus, χ^2^ showed insufficient fit. This statistic is known to be too restrictive, as it nearly always rejects the model when large samples are used. Regarding the other goodness-of-fit measures, RMSEA and SRMR values lower than 0.08 are considered indicative of a good fit, as are NFI, TLI, and CFI values higher than 0.90 (Bentler & Bonett, 1980). Figure 1 presents a graphical representation of the final 11-item factor structure model. The alternative models did not achieve satisfactory fit indices. In none of these models did the RMSEA achieve the required value lower than 0.80, and the other indicators (TLI, CFI, and NFI) a critical value higher than 0.90; see Fig. 1.

#### Reliability

Reliability of the 20-item and 11-positively worded item Polish SWBS was established with Cronbach’s alpha coefficient for the total score and subscales of Religious and Existential well-being; see Table S2 in Supplementary Material. For the 20-item SWBS, all Cronbach’s *α*s were ≥ 0.84. When the nine negatively worded items were excluded, all Cronbach’s *α*s were ≥ 0.85. So, the Polish SWBS achieved high internal consistency in both 20-item and 11-item versions.

#### Skew

The total score of the 20-item Polish SWBS was significantly negatively skewed in Sample 2 (Skew = -0.035, SE = 0.140, *p* = 0.006) but not in Sample 1 (Skew = 0.001, SE = 0.137, *p* = 0.234). Scores on the RWBS and EWBS had significantly negatively skewed distributions in both samples. The range of skew coefficients was from -0.357 to -0.148. The 11-item Polish SWBS had similar results; its total score was significantly negatively skewed in Sample 2 (Skew = -0.092, SE = 0.140, *p* = 0.032) but not in Sample 1 (Skew = 0.134, SE = 0.137, *p* = 0.465). Scores on its RWBS and EWBS had significantly negatively skewed distributions in both samples. The skew coefficients ranged from -0.335 to -0.155. Thus, the ceiling effect would be evidenced in the Polish SWBS (c.f., Bufford et al., 1991; Genia, 2001; Ledbetter et al., 1991; Scott et al., 1998).

#### Sociodemographic differences

The total score and the subscale scores of the 11-item Polish SWBS were compared among different sociodemographic groups in a pooled Sample 1 and 2 (*n* = 621). The scores were not normally distributed; however, a skew with an absolute value of less than 0.50 is considered sufficiently symmetrical (Slater et al., 2001). For this reason, parametric statistics were employed to test the differences. Gender differences were tested with a t-test and other comparisons with one-way ANOVA. Table 3 shows the results of the comparisons.

**Table 3:** Descriptive Statistics and Sociodemographic Differences of the Combined Data Set for Samples 1 and 2 (*n* = 621) for the Eleven-Item PSWBS

Variable		SWBS		RWB		EWB	
	*n* (%)	*M* (*SD*)	Significance	*M* (*SD*)	Significance	*M* (*SD*)	Significance
Total	621 (100)	3.49 (0.94)		3.71 (1.28)		3.23 (0.90)	
Gender							
Female	329 (53)	3.51 (0.97)	n.s.	3.70 (1.32)	n.s.	3.27 (0.91)	n.s.
Male	292 (47)	3.48 (0.91)		3.72 (1.22)		3.19 (0.89)	
Age, years							
1. 18–34	245 (39.5)	3.49 (0.98)	n.s.	3.72 (1.29)	n.s.	3.23 (0.97)	n.s.
2. 35–54	275 (44.3)	3.51 (0.95)		3.70 (1.28)		3.28 (0.88)	
3. 55 +	101 (16.3)	3.44 (0.93)		3.73 (1.23)		3.01 (0.74)	
Residence							
1. Village	162 (26.1)	3.33 (0.83)	*p* < .001 (1–3***, 2–3*), *η*^2^ = .02	3.42 (1.12)	*p* < .001 (1–3***, 2–3**) *η*^2^ = .04	3.21 (0.83)	n.s.
2. City below 100,000	270 (43.5)	3.45 (0.96)		3.64 (1.23)		3.22 (0.95)	
3. City above 101,000	189 (30.4)	3.69 (0.98)		4.05 (1.39)		3.26 (0.88)	
Marital status							
1. Single	125 (20.1)	3.49 (1.08)	n.s.	3.57 (1.40)	n.s.	3.40 (0.99)	*p* < .01 (1–4*, 2–4*), *η*^2^ = .04
2. Unmarried mate	143 (23.0)	3.63 (0.91)		3.87 (1.22)		3.35 (0.90)	
3. Divorced	46 (7.4)	3.63 (0.78)		3.84 (1.06)		3.37 (0.79)	
4. Married	296 (47.7)	3.41 (0.96)		3.69 (1.27)		3.08 (0.86)	
5. Widow/er	11 (1.8)	3.48 (0.95)		3.62 (1.43)		3.32 (0.80)	
Religiosity							
1. Religious	148 (23.8)	4.27 (0.75)	*p* < .001 (1–2***, 1–3***, 2–3*), *η*^2^ = .30	4.97 (1.02)	*p* < .001 (1–2***, 1–3***, 2–3*), *η*^2^ = .40	3.43 (0.93)	*p* < .001 (1–3***, 2–3*), *η*^2^ = .03
2. Neutral	312 (50.2)	3.47 (0.70)		3.64 (0.90)		3.26 (0.80)	
3. Non-religious	161 (25.9)	2.81 (0.98)		2.68 (1.10)		2.98 (0.99)	

The *p* value denotes all-group comparison, while results in parentheses denote multiple-group comparison with the Bonferroni correction;

n.s. non-significant; SWBS Spiritual Well-Being Scale; RWB Religious Well-Being; EWB Existential Well-Being, *η*^2^ = effect size

^***^p < .001; **p < .01; *p < .05

The Polish SWBS and its subscales did not show significant gender or age differences. In the residence groups, there were statistically significant differences in SWBS scores (*F*_2, 618_ = 7.07, *p* < 0.001, *η*^*2*^ = 0.02) and in RWBS scores (*F*_2, 618_ = 11.65, *p* < 0.001, *η*^*2*^ = 0.04). Tukey’s HSD Test for multiple comparisons found that the mean SWB of the people living in a city above 201,000 citizens (*M* = 3.69) was significantly higher than those living in a city with less than 200,000 citizens (*M* = 3.45, *p* = 0.018, 95% CI = [0.034, 0.451]) and those living in a village (*M* = 3.33, *p* < 0.001, 95% CI = [0.128, 0.598]). Similar differences were observed in RWB: the mean RWBS score of the people living in a city above 201,000 citizens (*M* = 4.05) was higher than those living in a city below 200,000 citizens (*M* = 3.64, *p* = 0.002, 95% CI = [0.126, 0.697]) and those living in a village (*M* = 3.42, *p* < 0.001, 95% CI = [0.306, 0.951]). No significant differences were found for EWB related to age or place of residence.

No statistically significant differences were found in the marital status groups in total SWB or RWB. However, differences were found in the Polish EWB subscale scores (*F*_2, 615_ = 3.79, *p* < 0.005, *η*^*2*^ = 0.02). Tukey’s HSD post hoc test showed that the mean EWB score of married participants (*M* = 3.08) was significantly lower than for single (*M* = 3.40, *p* = 0.011, 95% CI = [− 0.569, − 0.048]) and unmarried dating participants (*M* = 3.35, *p* = 0.046, 95% CI = [-0.500, -0.003]).

Finally, the Polish SWBS (*F*_2, 618_ = 129.36, *p* < 0.001, *η*^*2*^ = 0.30), RWBS (*F*_2, 618_ = 209.01, *p* < 0.001, *η*^*2*^ = 0.40) and EWBS (*F*_2, 618_ = 10.22, *p* < 0.001, *η*^*2*^ = 0.03) showed significant religiosity differences. Tukey’s HSD post hoc test indicated that the means on the SWBS (*M* = 4.27), RWBS (*M* = 4.97), and EWBS (*M* = 3.43) for religious people were significantly higher than the means on the SWBS (*M* = 3.47, *p* < 0.001, 95% CI = [0.614, 0.987]) and RWBS (*M* = 3.64, *p* < 0.001, 95% CI = [1.096, 1.559]) of religiously neutral people, and the means on SWB, RWB, and EWB of non-religious participants (*M* = 2.81, *p* < 0.001, 95% CI = [1.241, 1.666]; *M* = 2.68, *p* < 0.001, 95% CI = [2.027, 2.556]; *M* = 2.98, *p* < 0.001, 95% CI = [0.210, 0.684]), respectively.

#### Discussion – Study 1

Similar to other authors’ research in non-English speaking countries (e.g., Miller et al., 1998; Musa & Pevalin, 2014), our study did not replicate the two-factor structure in the 20-item Polish SWBS. EFA showed a three-factor structure, with a well-identified RWBS factor that included one of the four negatively worded items and two separate factors in EWB, the first formed by positively and the second by negatively worded items.

After removing the negatively worded items, an 11-item Polish SWBS was obtained with a good model fit for a two-factor structure. The reliability of the subscales and the total score of the SWBS is high, and the distributions of the scores are significantly left-skewed. Thus, it is possible that there is a ceiling effect, an overrepresentation of high scores, especially for religious people. A comparison of people with different demographic characteristics showed that scores on the Polish RWBS were higher among religious people and those living in big cities when compared to non-religious or religiously indifferent people and those living in small towns and villages. Higher EWBS scores were found in single and unmarried dating people than in married ones.

### Study 2. Correlates of the Polish SWBS

Study 2 aimed to analyze the relationships between the Polish SWBS and measures of positive and negative mood (GMS-PM, Wojciszke & Baryła, 2005), satisfaction with life (SWLS, Diener et al., 1985), anxiety (DBR-SIS, von der Embse et al., 2015), depression (BSD, Hakstian & McLean, 1989), and psychological well-being (PWBS, Ryff & Keyes, 1995).

We expected positive correlations between the Polish SWBS and positive mood, satisfaction with life, and psychological well-being, and negative correlations between the SWBS and negative mood, anxiety, and depression (Bufford & Paloutzian, 2023/4). Since the SWBS relates to religious and existential meanings, we expected it to correlate more strongly with cognitive than emotional well-being measures. Therefore, we expected a stronger correlation between the SWBS and SWLS than between the SWBS and mood, anxiety, and depression (Zarzycka et al., 2023). We also expected that RWB, which refers to a sense of well-being in a relationship with God, would correlate more strongly with cognitive (e.g., SWLS and PWBS) measures of well-being than EWB. Since it evokes existential references and relates to psycho-social processes, EWB was expected to correlate more strongly with emotional measures of well-being (positive and negative mood, anxiety, and depression) than RWB (Musa et al., 2018).

#### Method

##### Participants

Two samples of Polish adult college students were examined. Sample 3 consisted of 389 people (59.1% women) aged 18 to 45 (*M* = 20.72, *SD* = 3.14). Most of the respondents (*n* = 337, 86.6%) declared themselves to be Catholics. The remaining respondents represented other Christian denominations (e.g., Orthodoxy *n* = 20, 5.1%, Greek Catholicism *n* = 8, 2.1%, Protestants *n* = 1, 0.3%). Most respondents (*n* = 237, 60.7%) described themselves as religious, but in varying degrees. Specifically, some said they were definitely religious (*n* = 20, 5.1%), some as religious (*n* = 154, 39.6%), while others were moderately religious (*n* = 63, 16.2%). Other respondents described themselves as indifferent (*n* = 24, 6.2%), spiritual but non-religious (*n* = 47, 12.1%), not religious (*n* = 10, 2.6%), agnostic (*n* = 21, 5.4%), atheist (*n* = 41, 10.5%), and other (*n* = 5, 1.3%); the remainder did not respond (*n* = 4, 1.0%). Most participants (*n* = 181, 47.3%) reported living in major cities (with populations over 100.000) or villages (*n* = 111, 28.5%).

Sample 4 consisted of 101 adults (78.2% women) aged 29 to 86 (*M* = 44.34, *SD* = 8.93). Most of the respondents (*n* = 77, 76.2%) declared themselves Catholics, while others described themselves as none (*n* = 12, 11.9%). Respondents described themselves as definitely religious (*n* = 16, 15.8%), religious (*n* = 24, 23.8%), moderately religious (*n* = 25, 24.8%), indifferent (*n* = 12, 11.9%), or non-religious (*n* = 6, 5.9%); other described themselves as agnostic (*n* = 5, 5.0%) or atheist (*n* = 4, 4.0%); the remainder chosen option other (*n* = 9, 8.9%). Most of them (*n* = 80, 79.2%) reported living in cities above 100,000 inhabitants. The more detailed characteristics of respondents in Samples 3 and 4 are presented in Table S1 (see Supplementary Material).

##### Procedure

Samples 3 and 4 were recruited using a snowball method. Participants completed paper-based questionnaires. Correlations between the Polish SWBS and positive and negative mood (GMS-PM Wojciszke & Baryła, 2005), satisfaction with life (SWLS, Diener et al., 1985), anxiety (DBR-SIS, von der Embse et al., 2015), and depression (BSD, Hakstian & McLean, 1989) were established using Sample 3. Correlations between the SWBS and psychological well-being (PWBS, Ryff, 2014) were established using Sample 4. Anonymity was ensured, and informed consent was obtained from all participants. The procedure was approved by the Research Ethics Committee at the Institute of Psychology at the first author’s university.

##### Measures

*Spiritual Well-Being*. The initial 20-item Polish SWBS was administered to measure religious and existential well-being (Paloutzian & Ellison, 1982). Alpha coefficients were 0.90, 0.96, and 0.85 for the total SWBS, RWBS, and EWBS, respectively. However, based on the results of Study 1, all analyses in Study 2 were conducted on the Polish SWBS with 11 positively formulated items.

*General Mood.* The 10-item General Mood Scale (GMS) was used to measure Positive Mood (e.g., *I’m cheerful*) and Negative Mood (e.g., *I feel depressed*) (Wojciszke & Baryła, 2005). The response options ranged from 1 (*I don’t agree*) to 5 (*I agree*). The internal consistency of the GMS subscales in this study was alpha = 0.91 for both positive and negative moods (see Table 4).

*Satisfaction with Life.* The 5-item Satisfaction with Life Scale (SWLS) was used to measure respondents’ global cognitive judgments of satisfaction with life (e.g., *I am satisfied with my life, In most ways, my life is close to my ideal*) (Diener et al., 1985; Juczyński, 2001). The response options were from 1 (*strongly disagree*) to 7 (*strongly agree*). The internal consistency of the SWLS obtained in this study was 0.91 (see Table 4).

*Anxiety.* The 3-item Direct Behaviour Rating-Scale Items Scale (DBR-SIS) was applied to measure the respondents’ level of anxiety (von der Embse et al., 2015; Zarzycka et al., 2022). The DBR-SIS consists of three items on the Likert scale reflecting the social (*I am worried what others will think*), cognitive (*I feel restless*), and physiological (*I am nervous*) aspects of anxiety. The response options ranged from 1 (*no anxiety*) to 10 (*very high anxiety*). In this study, the internal consistency coefficient was 0.79 (see Table 4).

*Depression.* The 4-item Brief Screen for Depression (BSD) was applied to measure the level of depressive symptoms (e.g., *How many times during the last two days have you been preoccupied with thoughts of hopelessness, helplessness, pessimism, intense worry, unhappiness, and so on?*) (Hakstian & McLean, 1989; Zarzycka et al., 2022). Respondents rated the first item on a scale of 1 (*not at all*) to 5 (*all of the time*), with items 2–4 ranging from 1 to 10. The BSD is scored by summing the scores for items 2–4 and multiplying the item 1 score by 4, adding four times the item 1 score to produce an overall score. The BSD has fair internal consistency with alpha = 0.60 (see Table 4).

*Psychological Well-Being.* The 18-item Psychological Well-Being Scale (Karas and Cieciuch, 2017; Ryff, 2014) was used to measure people's psychological well-being (e.g., *When I look at the story of my life, I am pleased with how things have turned out*). Items are rated on a 6-point Likert scale from 1 (*strongly disagree*) to 6 (*strongly agree*). The internal consistency of the PWBS obtained in this study was 0.68 (see Table 4).

#### Data Analysis

We calculated Pearson’s correlations between the study variables and then Lee and Preacher’s (2013) procedure to test the difference between two dependent correlations with one variable in common.

#### Results

Table 4 shows the Pearson correlations for the Study 2 variables. The Polish SWBS subscales had a small positive correlation with each other (*r* = 0.30, *p* < 0.001). The RWBS (*r* = 0.91, *p* < 0.001) correlated more strongly positively with the total score of the SWBS than EWBS (*r* = 0.68, *p* < 0.001); these relationships were medium and large respectively. As hypothesized, the total SWBS score correlated positively with positive mood and satisfaction with life and negatively with negative mood, anxiety, and depression. The RWBS subscale had a small positive correlation with the SWLS but did not correlate with mood (neither positive nor negative), depression, or anxiety. The EWBS had medium positive correlations with positive mood and satisfaction with life and negative correlations with anxiety, depression, and negative mood (see Table 4). We also calculated correlations for the 20-item SWBS. The correlation matrix is included in Table S3 (see Supplementary Material).

**Table 4:** Intercorrelations, Means, Standard Deviations, and Alphas for the Variables of Interest in Samples 3 and 4 for the 11-Item Polish Spiritual Well-Being Scale

Variable		1	2	3	4	5	6	7	8	9
1	SWB^a^	–								
2	RWB^a^	0.91 ***	–							
3	EWB^a^	0.68 ***	0.30 ***	–						
4	GMS-P^a^	0.27 ***	0.02	0.58 ***	–					
5	GMS-N^a^	− 0.32***	− 0.10	− 0.55 ***	− 0.81 ***	–				
6	SWLS^a^	0.51 ***	0.26 ***	0.71 ***	0.48 ***	0.46***	–			
7	BDR-SIS^a^	− 0.18***	0.01	− 0.42 ***	− 0.56 ***	0.60 ***	− 0.33***	–		
8	BSD^a^	− 0.23***	− 0.03	− 0.47 ***	− 0.55 ***	0.55 ***	− 0.39 ***	0.54***	–	
9	PWBS^b^	0.29 ***	0.07	0.69 ***	–	–	–	–	–	–
	*M*	3.64	3.78	3.96	3.29	2.34	3.96	4.78	24.85	4.76
	*SD*	1.01	1.44	0.97	1.01	1.05	1.10	2.21	8.21	0.30
	*Skew*	− 0.14	− 0.19	− 0.45	− 0.38	0.54	− 0.05	0.23	0.15	− 0.11
	Alpha	0.90	0.96	0.85	0.91	0.91	0.79	0.79	0.60	0.68

SWBS Spiritual Well-Being Scale; RWB Religious Well-Being; EWB Existential Well-Being; GMS-P General Nood Scale-Positive Mood; GMS-N General Mood Scale-Negative Mood; SWLS Satisfaction with Life Scale; BDR-SIS Direct Behavior Rating-Scale Items Scale; BSD Brief Screen for Depression; PWBS Psychological Well-Being Scale.

^a^Analyses were carried out on Sample 3, *N* = 389

^b^Analyses were carried out on Sample 4, *N* = 101

^***^ *p* < .001

Considering Lee and Preacher’s (2013) procedure for calculation for the test of the difference between two dependent correlations with one variable in common, we found that the correlations between SWBS and life satisfaction were stronger than those between SWBS and affect indicators, that is, positive (z-test = 3.84, *p* < 0.001), negative (z-test = 3.09, *p* < 0.001) mood, anxiety (z-test = 5.18, *p* < 0.001) and depression (z-test = 4.44, *p* < 0.001). These results may suggest that spiritual well-being is more related to cognitive than affective constructs.

#### Discussion – Study 2

As expected, the Polish SWBS subscales had a positive, though small, correlation with each other, meaning that they measure different aspects of well-being. They positively correlated with the SWBS total score, but the RWBS correlated more strongly than the EWBS. Thus, SWB is more closely related to a religious perspective than an existential perspective in the Polish context (CBOS, 2020; Zarzycka, 2009). The pattern of correlations with measures of well-being was also as expected. The SWBS correlated positively with SWLS, positive mood, and psychological well-being and negatively with depression, anxiety, and negative mood. The SWBS was more positively related to SWLS, a cognitive measure of well-being than emotional measures. The EWBS correlated more strongly with SWLS than SWB. Mood, anxiety, and depression also correlated more strongly with the EWBS than with the RWBS, which further confirms the validity of the construct under study. These results are generally consistent with those reported by Bufford and Paloutzian (2023/4).

## General Discussion

In this study, we assessed the internal structure and psychometric properties of the SWBS in a Polish, predominantly and culturally Catholic sample. We also examined how spiritual well-being is related to mental health. The EFA revealed a three-factor structure of the 20-item Polish SWBS, which is different from the original two-factor structure (Ellison, 1983). As expected, items that were positively worded (PWI) formed RWBS and EWBS factors. The problem arose with eight of nine items that were negatively worded (NWI). The NWI that originally belonged to EWBS made a separate factor, whereas those originally belonging to RWBS were cross-loaded on two factors, with lower loadings for F3 in the PRWB. Varimax (Ellison, 1983) and oblique rotation (Ledbetter et al., 1991) produced similar results.

The CFA confirmed the best model fit for the 11-item two-factor structure containing only positively-worded items. We also confirmed the internal consistency of the 11-item PSWBS. The often-reported left skew of SWBS scores was also confirmed. Thus, there is a ceiling effect; that is, the Polish SWBS is also less sensitive to differentiating high levels of SWB. The associations of PSWBS with health indicators were as expected: positive with SWLS, positive mood, and psychological well-being, and negative with depression, anxiety, and negative mood: these relationships tended to be stronger for EWB than for RWB. Thus, the eleven-item Polish SWBS that includes only positively worded items is a reliable and accurate method for measuring spiritual well-being among Polish adults and can be used by researchers and clinicians. Due to the differences in the number of items in the Polish SWBS compared to other versions of the scale, we propose to use the mean item score rather than the sum for the total score and subscale scores. This will allow comparisons of results obtained in different cultural and linguistic backgrounds with varying numbers of items.

The methodological factor associated with NWI appeared not only in the Polish SWBS but also in several other SWBS translations (Musa & Pevalin, 2014; Tavel et al., 2022; You & Yu, 2016) as well as in other scales measuring spiritual well-being, for example, Functional Assessment of Chronic Illness Therapy-Spiritual Well-Being (Šarníková et al., 2018; Tavel et al., 2022). The two-factor structure of SWBS, composed of PWIs, was confirmed in a Czech, highly secularized population (Malinakova et al., 2017; Tavel et al., 2022). Interpreting the results, the authors (Malinakova et al., 2017) concluded that in a highly secular environment such as the Czech Republic, it is possible that respondents’ choice of the “I strongly disagree” option of negatively worded RWBS items express their disapproval of the way the items were worded since the items implicitly assumed the existence of God or religious beliefs. In Polish, a religious, Catholic context, negatively worded items may be understood as an indicator of spiritual strain, whether religious, existential, or both. When religious people respond to items such as “I do not get much personal strength and support from my God” or “I feel that life is full of conflict and unhappiness,” they may manifest strain rather than just the absence or low levels of well-being. If religiousness is no longer serving as a resource, it may begin to be experienced as a source of stress. Religious people often do not admit to experiencing religious struggles, or they deny them because they consider them morally wrong. This is confirmed by extensive research on religious struggles (Zarzycka, 2023). Bufford and Paloutzian (2023/4) related the methodological factor of NWI to linguistic and cultural aspects, particularly as the EWBS items contain no reference to God and thus do not seem sensitive to religious strain. Following the recommendation from other studies (e.g., Malinakova et al., 2017; Tavel et al., 2022) we suggest that the Polish SWBS is limited to eleven positively worded items.

The left-skew of the SWBS results suggests the presence of a ceiling effect. Thus, the Polish version may, perhaps, with less accuracy, differentiate people with high levels of spiritual well-being. This is a typical result in studies using the SWBS in general Judeo-Christian samples (Bufford et al., 1991; Genia, 2001; Ledbetter et al., 1991; Scott et al., 1998).

The reliability of the Polish EWB subscale is lower than that of the RWB subscale, which aligns with the results obtained by other authors (Ellison, 1983; Tavel et al., 2021). Tavel et al. (2021) explained this difference by the greater heterogeneity of the existential well-being construct. RWBS items focus on the relationship with God, whereas EWBS items cover different areas of a person’s existence, such as life goals, life satisfaction, and vision for the future. The correlation between religious and existential well-being (*r* = 0.30) supported the finding that they are related but distinct constructs (e.g., Genia, 2001; Paloutzian & Ellison, 1982; Tavel et al., 2022). Therefore, it is reasonable to report subscale scores (Genia, 2001), while more ambiguity may characterize the interpretation of the global SWBS score.

Stronger correlations between the Polish RWBS (as compared to the EWBS) and cognitive measures of well-being (e.g., the SWLS), and between the Polish EWBS (as compared to the RWBS) and emotional indicators of well-being (e.g., anxiety and depression), are also consistent with prior results that confirmed the role of religion in meaning-making processes (Park, 2013). A comparison of people with different demographic characteristics regarding spiritual well-being showed that RWB is higher among Polish people living in large cities than those in small towns and villages. Lower EWB is found among married people than single and unmarried dating people. Religious people had higher RWB than non-religious and religiously indifferent people, whereas non-religious people had lower EWB than neutral people and believers.

### Limitation

This study has several limitations. First, we did not examine the validity of the scale to religiousness/spirituality. Future research would benefit from establishing the convergent and differential validity of the SWBS scale in relation to methods that measure religious and spiritual constructs, such as religious and spiritual comfort and struggle (Falewicz et al., 2023; Zarzycka, 2014). Second, analyses of the test–retest reliability of the scale are desirable. Well-being is a fluctuating variable; therefore, the stability of responses given to scale items requires wider attention in separate studies. Third, a limitation of the current study is that it uses self-report measures only, which does not allow us to rule out self-serving bias or areas of unawareness of participants’ personal characteristics. Fourth, participants were from the general population, but we did not test the structure and psychometric properties of the Polish SWBS in clinical samples. Using clinical samples in future studies could help illuminate differences in SWBS properties or utility among more specialized Polish sub-populations. Finally the present study used convenient samples that may not accurately represent the Polish adult population. Despite these limitations, the results indicate that the Polish SWBS can be an effective measure of spiritual well-being that is helpful for both researchers and clinicians.

## Conclusion

This article presents the results of two studies confirming the successful validation of the Polish version of the SWBS, which can be used to assess spiritual well-being. We found that the Polish version of the SWBS scale contains 11 positively worded items (6 RWBS and 5 EWBS), shows satisfactory reliability, and can assess religious and existential well-being in adults. Moreover, the study contributes to understanding spiritual well-being in the context of psychological well-being.

## Supplementary Information

Below is the link to the electronic supplementary material.

Supplementary file1 (DOCX 84 KB)

## Data Availability

The data that support the findings of this study are openly available in the OSFHOME Repository at https://osf.io/m2tfb/?view_only=bf948be3dca04460bc09e0f0dd4f830c
