# Supplementary material for: The Spiritual Well-Being Scale in the Polish Catholic Setting: Construct, Structure, and Relationships with Psychological Well-Being
Source: J Relig Health. 2024 Oct 6;63(6):4191–214. doi: 10.1007/s10943-024-02138-w (PMC11576776; doi:10.1007/s10943-024-02138-w)
Supplement: Supplementary file 1 — Supplementary file1 (DOCX 84 KB) [file 10943_2024_2138_MOESM1_ESM.docx]

**Table S1**

Participant Demographic Characteristics

| Variable | Study 1 | | Study 2 | |
| --- | --- | --- | --- | --- |
|  | Sample 1  *N* = 318 | Sample 2  *N* = 303 | Sample 3  *N* = 389 | Sample 4  *N* = 101 |
| Sex, *n* (%) |  |  |  |  |
| 1. Women | 165 (51.9%) | 164 (54.1%) | 230 (59.3%) | 79 (78.2%) |
| 2. Men | 153 (48.1%) | 139 (45.9%) | 158 (40.7%) | 22 (21.8%) |
| Confession, *n* (%) |  |  |  |  |
| 1. Roman Catholic | 267 (84.0%) | 229 (75.6%) | 337 (86.6%) | 77 (76.2%) |
| 2. Greek Catholicism | 3 (0.9%) | 6 (2.0%) | 8 (2.1%) | 1 (1.0%) |
| 3. Protestants | 2 (0.6%) | - | 1 (0.3%) | 1 (1.0%) |
| 4.Orthodoxy | 1 (0.3%) | - | 20 (5.1%) | 1 (1.0%) |
| 5. Other | 5 (1.5%) | 14 (4.6%) | 16 (4.1%) | 9 (9.0%) |
| 6. None | 40 (12.6%) | 54 (17.8%) | 3 (0.8%) | 12 (11.9%) |
| Religiosity, *n* (%) |  |  |  |  |
| 1. Definitely religious | 12 (3.8%) | 17 (5.6%) | 20 (5.1%) | 16 (15.8%) |
| 2. Religious | 62 (19.5%) | 70 (23.1%) | 154 (39.6%) | 24 (23.8%) |
| 3. Moderately religious | 111 (34.9%) | 86 (28.4%) | 63 (16.2%) | 25 (24.8%) |
| 4. Moderately non-religious | 57 (17.9%) | 58 (19.1%) | - | - |
| 5. Non-religious | 33 (10.4%) | 27 (8.9%) | 10 (2.6%) | 6 (5.9%) |
| 6. Definitely non-religious | 43 (13.5%) | 45 (14.9%) | - | - |
| 7. Indifferent | - | - | 24 (6.2%) | 12 (11.9%) |
| 8. Spiritual but non-religious | - | - | 47 (12.1%) | - |
| 9. Agnostic | - | - | 21 (5.4%) | 5 (5.0%) |
| 10. Atheist | - | - | 41 (10.5%) | 4 (4.0%) |
| 11. Other | - | - | 5 (1.3%) | 9 (8.9%) |
| 12. None | - | - | 4 (1.0%) | - |
| Residence, *n* (%) |  |  |  |  |
| 1. Village | 83 (26.1%) | 79 (26.0% | 111 (28.5%) | 7 (6.9%) |
| 2. City below 100,000 | 151 (47.7%) | 119 (39.1%) | 90 (23.5%) | 14 (11.2%) |
| 3. City over 100,001 | 84 (26.4%) | 105 (34.5%) | 181 (47.3%) | 80 (79.2%) |
| Education, *n* (%) |  |  |  |  |
| 1. Elementary | 6 (1.9%) | 10 (3.3%) | - | - |
| 2. Vocational | 28 (8.8%) | 21 (6.9%) | - | - |
| 3. Secondary | 136 (42.8%) | 132 (43.4%) | - | - |
| 4. Student | 15 (4.7%) | 8 (2.6%) | - | - |
| 5. High | 133 (41.8%) | 132 (43.4%) | - | - |
| Marital status, *n* (%) |  |  |  |  |
| 1. Single | 61 (18.6%) | 64 (21.1%) | - | - |
| 2. Unmarried mate | 66 (20.1%) | 77 (24.8%) | - | - |
| 3. Divorced | 24 (7.5%) | 22 (7.3%) | - | - |
| 4. Married | 161 (50.6%) | 135 (44.6%) | - | - |
| 5. Widow/er | 6 (1.9%) | 5 (1.3%) | - | - |

**Table S2**

Descriptive statistics and reliability of 20 and 11-item Polish Spiritual Well-Being Scale

|  | *M* | *SD* | *Skew* | *Alpha* |
| --- | --- | --- | --- | --- |
| Sample 1 (*N* = 318) |  |  |  |  |
| *20-item version* |  |  |  |  |
| SWBS | 3.61 | 0.73 | 0.001 | .88 |
| RWB | 3.43 | 1.04 | -0.183 | .90 |
| EWB | 3.80 | 0.75 | -0.357 | .84 |
| *11-item version* |  |  |  |  |
| SWBS | 3.51 | 0.91 | 0.134 | .90 |
| RWB | 3.29 | 1.24 | -0.167 | .94 |
| EWB | 3.77 | 0.89 | -0.285 | .85 |
| Sample 2 (*N* = 303) |  |  |  |  |
| *20-item version* |  |  |  |  |
| SWBS | 3.63 | 0.79 | -0.035 | .90 |
| RWB | 3.45 | 1.12 | -0.148 | .92 |
| EWB | 3.81 | 0.76 | -0.277 | .84 |
| *11-item version* |  |  |  |  |
| SWBS | 3.50 | 0.97 | -0.092 | .92 |
| RWB | 3.29 | 1.32 | -0.155 | .95 |
| EWB | 3.76 | 0.91 | -0.335 | .85 |

*Note*. SWBS – Spiritual Well-Being; RWB – Religious Well-Being;
EWB – Existential Well-Being.

**Table S3**

*Intercorrelations, Means, Standard Deviations, and Alphas for the Variables of Interest in Samples 3 and 4 for the 20-item Polish Spiritual Well-Being Scale*

| Variable | | 1 | 2 | 3 | 4 | 5 | 6 | 7 | 8 | 9 |
| --- | --- | --- | --- | --- | --- | --- | --- | --- | --- | --- |
| 1 | SWB^@^ | ⎯ |  |  |  |  |  |  |  |  |
| 2 | RWB^@^ | .88 *** | ⎯ |  |  |  |  |  |  |  |
| 3 | EWB^@^ | .76 *** | .36 *** | ⎯ |  |  |  |  |  |  |
| 4 | GMS-P^@^ | .31 *** | .02 | .58 *** | ⎯ |  |  |  |  |  |
| 5 | GMS-N^@^ | -.37*** | -.10 | -.59 *** | -.81 *** | ⎯ |  |  |  |  |
| 6 | SWLS^@^ | .54 *** | .25 *** | .72 *** | .48 *** | .46*** | ⎯ |  |  |  |
| 7 | DBRSI^@^S | -.21*** | .02 | -.45 *** | -.56 *** | .60 *** | -.33*** | ⎯ |  |  |
| 8 | BSD^@^ | -.25*** | -.01 | -.48 *** | -.55 *** | .55 *** | -.39 *** | .54*** | ⎯ |  |
| 9 | PWBS^ | .30 *** | .05 | .68 *** | ⎯ | ⎯ | ⎯ | ⎯ | ⎯ | ⎯ |
|  | *M* | 3.77 | 3.59 | 3.94 | 3.29 | 2.34 | 3.96 | 4.78 | 24.85 | 4.76 |
|  | *SD* | 0.89 | 1.25 | 0.89 | 1.01 | 1.05 | 1.10 | 2.21 | 8.21 | 0.30 |
|  | *Skew* | -0.03 | -0.16 | -0.43 | -0.38 | 0.54 | -0.05 | 0.23 | 0.15 | -0.11 |
|  | Alpha | .91 | .92 | .88 | .91 | 0.91 | .79 | .79 | .60 | .68 |

*Note*. SWBS – Spiritual Well-Being Scale; RWB – Religious Well-Being; EWB – Existential Well-Being; GMS-P – General Mood Scale-Positive Mood; GMS-N – General Mood Scale-Negative Mood; SWLS - Satisfaction with Life Scale; DBR-SIS – Direct Behavior Rating – Scale Items Scale; BSD – Brief Screen for Depression; PWBS – Psychological Well-Being Scale.

*** *p* < .001

^@^ Analyses were carried out on Sample 3, N= 389.

^ Analyses were carried out in Sample 4, *N* = 101.
